# Erectile dysfunction in obstructive sleep apnea patients: A randomized trial on the effects of Continuous Positive Airway Pressure (CPAP)

**DOI:** 10.1371/journal.pone.0201930

**Published:** 2018-08-08

**Authors:** Mercè Pascual, Jordi de Batlle, Ferran Barbé, Anabel L. Castro-Grattoni, Josep M. Auguet, Lydia Pascual, Manel Vilà, Anunciación Cortijo, Manuel Sánchez-de-la-Torre

**Affiliations:** 1 Urology Department, Hospital Universitari Santa Maria, Lleida, Spain; 2 Group of Translational Research in Respiratory Medicine, Hospital Universitari Arnau de Vilanova and Santa Maria, IRBLLEIDA, Lleida, Spain; 3 Centro de Investigación Biomédica en Red de Enfermedades Respiratorias (CIBERES), Madrid, Spain; University of Sydney, AUSTRALIA

## Abstract

**Objectives:**

Obstructive sleep apnea (OSA) is among the least studied risk factors for erectile dysfunction (ED). We aimed to determine ED prevalence in newly-diagnosed OSA patients, describe their main characteristics and assess continuous positive airway pressure (CPAP) effects on ED.

**Methods:**

Cross-sectional study assessing ED prevalence in OSA patients and open-label, parallel, prospective randomized controlled trial evaluating 3-month CPAP treatment effects on sexual function, satisfaction, and psychological, hormonal and biochemical profiles. Male patients newly diagnosed with moderate/severe OSA (apnea-hypopnea index >20 events·h^−1^), aged 18–70 years, attending the sleep unit of a Spanish hospital during 2013–2016 were considered. A total of 150 patients were recruited (75 randomized ED patients). ED was defined as scores <25 on International Index Erectile Function 15 test. Wilcoxon’s matched-pairs signed-ranks and rank-sum tests were used.

**Results:**

ED prevalence was 51%. Patients with ED were older (p<0.001), had greater waist-to-hip ratios (p<0.001), were more frequently undergoing pharmacological treatment (p<0.001) and had higher glucose levels (p = 0.024) than non-ED patients. Although significant increases in erectile function (mean(SD) change: +4.6(7.9); p = 0.002), overall satisfaction (+1(2.2); p = 0.035), and sexual satisfaction (+2.1(4.3); p = 0.003) were found after CPAP treatment, only differences in sexual satisfaction (p = 0.027) and erectile function (p = 0.060) were found between study arms. CPAP treatment did not impact psychological, hormonal or biochemical profiles.

**Conclusions:**

This study confirmed the relationship between OSA and ED, suggesting the potential usefulness of ED screening in OSA patients, but could not determine conclusively whether CPAP is an effective stand-alone ED treatment, regardless of positive results on sexual satisfaction.

**Trial registration:**

ClinicalTrials.gov NCT03086122

## Introduction

Obstructive sleep apnea (OSA), defined as the presence of repetitive episodes of upper airway collapse during sleep, is a common chronic condition affecting 10% of middle-aged men [1]. Although OSA can be asymptomatic, it is usually characterized by intermittent hypoxia, daytime sleepiness, and an overall decrease in quality of life. OSA has been related to many pathologies, such as cardiovascular diseases [2], stroke [3] and hypertension [4]. Interestingly, the vascular implications of recurrent airway obstruction, intermittent hypoxemia and arousal from sleep, together with OSA-induced hormonal and psychological changes, have been suggested to increase the risk for erectile dysfunction (ED) [5–7].

ED, defined as the persistent inability to attain and maintain an erection sufficient to allow for satisfactory sexual performance [8], is a frequent phenomenon estimated to be present in 20% of men aged 30–70 years old, with a steep age-related increase in prevalence [9–11]. However, ED prevalence has been described as being as high as 69% among OSA patients [7], and in such patients, ED is considered to be a risk factor for future cardiovascular complications. Few studies to date have assessed whether continuous positive airway pressure (CPAP) treatment, one of the therapeutic options for OSA, could improve ED in OSA patients. Two reviews of the subject analyzing data through December 2014 concluded that although CPAP could improve sexual function and sexual satisfaction, randomized controlled trials (RCTs) were needed to confirm these potential benefits [12,13].

To increase knowledge on OSA-related ED, we first designed a cross-sectional study assessing ED prevalence in newly diagnosed OSA patients, comparing the hormonal, biological and psychological profiles of OSA patients with and without ED. Next, with the hypothesis that CPAP treatment could effectively reverse OSA-related ED, improving sexual function, hormonal profiles and sexual satisfaction, we designed an RCT randomizing OSA patients with ED to receive CPAP treatment or not.

## Methods

### Study design and population

Cross-sectional study considering consecutive male patients newly diagnosed with moderate to severe OSA, aged 18 to 70 years and in a current relationship who were attending the sleep unit of Hospital Univ Arnau de Vilanova-Santa Maria, Spain, between April 1^st^ 2013 and July 31^th^ 2016. Followed by an open-label, parallel, prospective RCT, including only those OSA patients that were diagnosed of ED, with a 3-month follow-up ending between September 1^st^ 2013 and September 30^th^ 2016.

Exclusion criteria: previous CPAP treatment; previously diagnosed sleep disorders; patients with >50% central apneas; Cheyne-Stokes respiration; daytime sleepiness as measured by the Epworth Sleepiness Scale (ESS) score >10 (S1 Questionnaires); psychophysical inability to complete questionnaires; uncontrolled or advanced chronic diseases (cardiovascular disease, multiple sclerosis, Parkinson's disease, spinal disc disease, schizophrenia, bipolar disorder, chronic renal failure and inflammatory bowel disease); pelvic surgery (radical prostatectomy); congenital or acquired malformations (Peyronie's disease, hypospadias, epispadias, and penile fracture); hormonal alterations (hypogonadism, hyperprolactinemia, hyper- or hypothyroidism, and Cushing's disease); and drug abuse (alcohol, cocaine, and heroin).

OSA patients fulfilling inclusion criteria were referred to the Urology Department, where they were informed about the project and asked to participate. Recruited patients were assessed for ED and answered to the study questionnaires. Web-based randomization of the ED patients (non-ED patients did not participate in the RCT) was performed using a secure, automated, password-protected system based on a permuted block design with a computer random number generator and a fixed block size of 10. Allocation was revealed only at the time of each subject randomization. No blinding measures were implemented. The ED patients were randomly assigned at a 1:1 ratio to immediately begin CPAP treatment (ED CPAP group) or to postpone CPAP treatment for 3 months (ED non-CPAP group). Afterwards, the recruited patients returned to the sleep unit for additional measurements and CPAP titration when required. All of the patients included in the RCT were followed up for 3 months and were reassessed in terms of ED-related clinical, biological and psychological aspects.

The ethics committee of Hospital Univ Arnau de Vilanova approved the study (CEIC-996; Mar. 27^th^ 2012), and all patients provided written informed consent. The RCT was registered at ClinicalTrials.gov (NCT03086122). Unfortunately, although the authors believed the trial to be registered on 2012, a successful registration was not achieved before the inclusion of the first patient due to administrative mistakes. Nevertheless, this study was performed according to the protocol submitted for ethical review on Mar. 2012. The authors confirm that all ongoing and related trials for this intervention are registered.

### Data collection

OSA was defined by means of a valid complete polysomnography reporting an apnea–hypopnea index (AHI) >20 events·h^−1^ with ≤50% of central apneas, according to the Spanish Respiratory Society (SEPAR) guidelines [14]. Obstructive apnea was defined as an absence or reduction (>90%) of airflow lasting ⩾10 s in presence of abdominal and thoracic movements. Obstructive hypopnoea was defined as a reduction (30% to 90%) in airflow lasting ⩾10 s associated with a decrease in arterial oxygen saturation ⩾3% and/or a micro-awakening on the electroencephalogram, in presence of both thoracic and abdominal movements. The AHI was defined as the number of episodes of apnea and hypopnea per hour of recording. Central apneas were defined as an absence airflow lasting at least 10 s in absence of thoracic and abdominal wall movements. A minimum of 3 hours of satisfactory signal recording was required to consider the test as valid.

CPAP titration was performed using an auto-CPAP device (Autoset-T, ResMed, Sydney, Australia) as previously validated by the Spanish Sleep and Breathing Group [15]. Up to two additional night recordings were attempted if the original recording was unacceptable. The determination of the optimal pressure was assessed by visually analyzing the pressure curve, which included the periods with a leak lower than 0.4 L/s (90^th^ centile). At-home treatment was initiated with a fixed CPAP level after the optimal pressure was determined.

ED was assessed by means of the International Index Erectile Function 15 (IIEF15) test [16], in its Spanish version [17], which allows for the assessment of sexual function broadly encompassing erectile function, orgasmic function, sexual desire, sexual satisfaction and overall satisfaction (S1 Questionnaires). The test was administered by a Urology department physician. Patients were classified as having ED when their erectile function domain score was less than 25 points.

Sociodemographic variables, as well as lifestyle habits, clinical background and usual pharmacological treatment, were recorded using questionnaires. Anthropometric measurements included weight; height; body mass index (BMI); neck, waist and hip circumferences; and waist to hip ratio (WHR). Fasting blood samples were obtained between 8:00h and 9:00h a.m. in the span of few days between recruitment and polysomnography. Serum and plasma samples were stored at -80°C. Biochemical profiling included glucose, lipids (triglycerides, total cholesterol, and high-density lipoprotein **(**HDL) and low-density lipoprotein (LDL) cholesterol), creatinine, urate, sodium, potassium, proteins, glutamyl pyruvic transaminase (GPT), gamma-glutamyl transferase (GGT) and C-reactive protein (CRP). Hormonal profiling included total testosterone, free testosterone, sex hormone binding globulin (sHBG), prolactin, luteinizing hormone (LH) and follicle-stimulating hormone (FSH), all measured by RIA (R&D Systems Europe, Abingdon, UK). Additionally, a basic hemogram was performed. Blood pressure was routinely measured. Continuous recording of night-time oxygen saturation by means of pulse oximetry was conducted routinely at the time of polysomnography. Finally, the Self-esteem and Relationship (SEAR) test [18], a self-administered test designed to specifically evaluate psychosocial aspects associated with ED, was administered to all patients during their Urology Department visits (S1 Questionnaires). The patients included in the RCT underwent reassessments of IIEF15 and SEAR, biochemical and hormonal profiles and CPAP-related variables after 3 months (in the CPAP arm), during a follow-up visit. Fasting blood samples were obtained between 8:00h and 9:00h a.m. on the same day of the follow-up visit.

### Statistical analyses

Means ± standard deviation (SD), medians (IQR) or frequencies (%) were computed to evaluate the differences between OSA patients with and without ED, assessing the significance of such differences with the chi-square test, independent samples Student’s t test or Mann-Whitney U test, as appropriate. The effects of CPAP treatment in the RCT were assessed on an intention to treat basis by comparing the differences between baseline and 3-month measurements using Wilcoxon’s matched-pairs signed-ranks test. The comparison of changes between non-CPAP and CPAP patients was performed by means of Wilcoxon’s rank-sum test. Finally, as a complementary analysis, multivariate ordered logistic regression models were constructed to ensure that key variables differing between ED and non-ED patients were not confounding the results on CPAP treatment effect.

Accepting an alpha risk of 0.05 and a beta risk of 0.2 in a two-sided test, 63 subjects were necessary in each RCT study arm to recognize as statistically significant a 2-unit difference in the erectile function score (IIEF15) after the intervention. The 2-unit minimal clinically important difference was defined according to the results by Rosen et al for mild ED patients [19]. The common SD was assumed to be 4 units. Assuming a 69% prevalence of ED among OSA patients [7,20], 183 OSA patients needed to be assessed to obtain the required 126 ED patients. However, although having a randomization goal of 126 ED patients, the study was stopped at 75. The main reasons for not reaching the recruitment goal were, first, the lower-than-expected prevalence of ED coupled with tight inclusion criteria, causing a very slow recruitment pace (a planned 2-year study turned out having a 3.5 years recruitment phase); second, the financial constrains caused by the extended recruitment phase; and third, the need to avoid biases caused by changes in the personnel assessing the patients.

Data analysis was conducted using Stata software, version 12.1 (StataCorp, College Station, TX, USA). The threshold for significance was set at 0.05.

## Results

**Fig 1:**
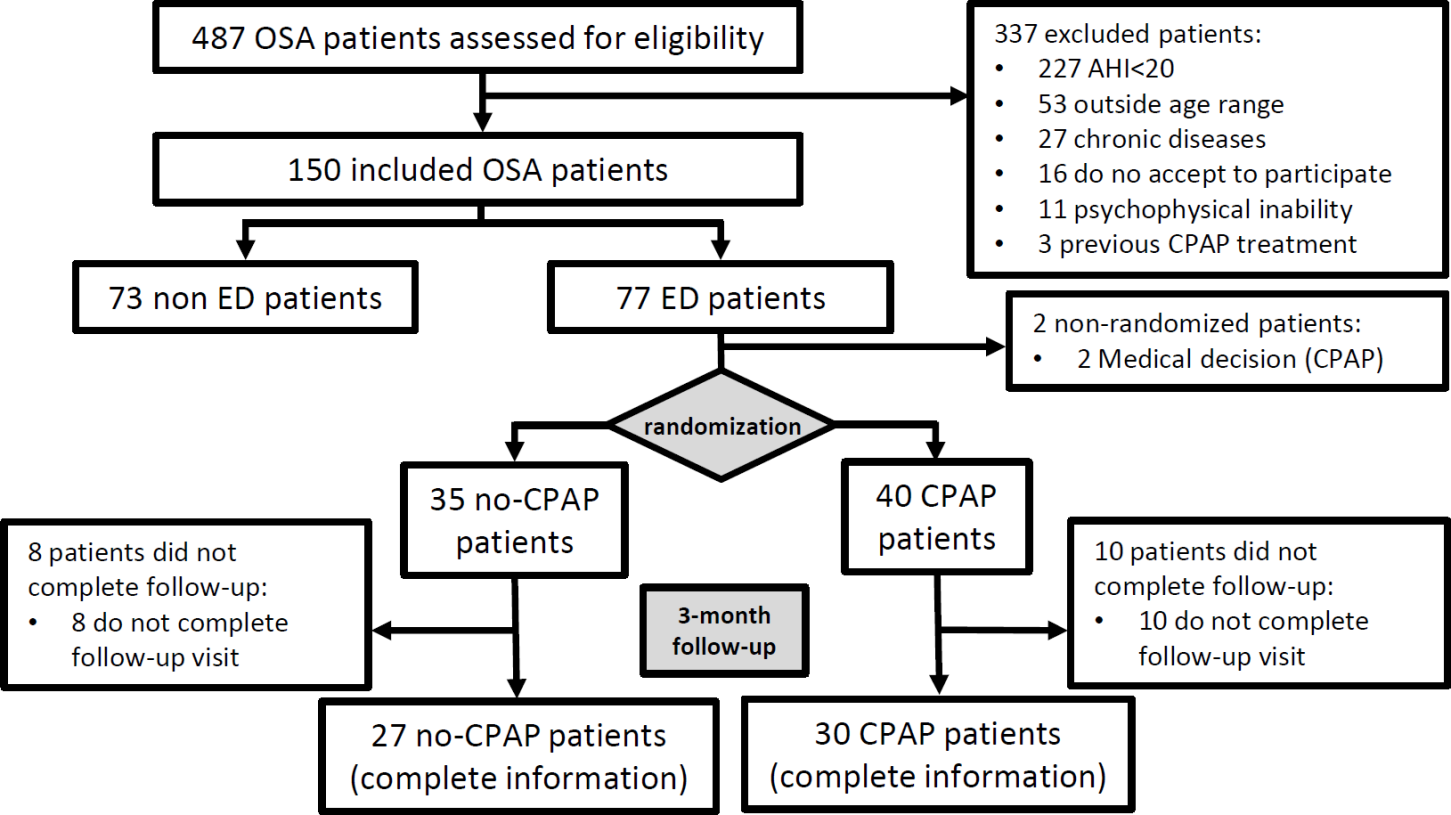
Study flowchart.

Up to 487 newly diagnosed male OSA patients were screened. After excluding patients who did not meet the inclusion criteria, a total of 150 patients (77 with ED and 73 without ED) were included in the analyses, and 75 ED patients were randomized (Fig 1).

The prevalence of ED among the OSA patients in our study was 51% (95% CI: 43%-60%).Table 1 shows the main characteristics of the OSA patients according to ED presence. ED patients were older, had greater WHRs and were more frequently undergoing pharmacological treatment than patients without ED. None of the patients reported use of Type 5 phosphodiesterase inhibitors. No differences in hormonal or biochemical profiles were found, except for higher glucose levels in ED patients (Table 2). Finally, Table 3 shows IIEF15 and SEAR scores. The median (IQR) erectile function score for non-ED patients was 29 (27–30), and the total SEAR score was 78 (71–86). In contrast, ED patients had an erectile function score of 18 (6–22) and a SEAR score of 62 (50–77).

**Table 1:** Main characteristics of OSA patients according to erectile dysfunction (ED) presence.

	non-ED n = 73	ED n = 77	p-value*
Age, years	48.7 (1.1)	54.8 (1)	**<0.001**
Body mass index, kg·m^−2^	32.2 (0.6)	33.0 (0.6)	0.274
Waist to hip ratio	0.98 (0.01)	1.04 (0.01)	**<0.001**
Systolic blood pressure, mm Hg	135 (2)	138 (2)	0.271
Diastolic blood pressure, mm Hg	88.3 (1.2)	88.7 (1.4)	0.844
AHI, events·h^−1^	46.1 (20.1)	51.6 (20.9)	0.121
Mean SaO_2_, %	74.4 (9.6)	71.9 (12)	0.174
Minimum SaO_2_, %	90.5 (9.1)	92.5 (8)	0.171
Time with SaO_2_ <90%, %	20.2 (21.6)	19.4 (21.5)	0.829
Epworth Sleepiness Scale	9.6 (5.3)	10.2 (5.8)	0.556
Nycturia, n (%)	49 (74%)	49 (72%)	0.776
Employment status, n (%)			**0.018**
Active	57 (83%)	48 (66%)	
Inactive	9 (13%)	11 (15%)	
Retired	3 (4%)	14 (19%)	
Stable partner, n (%)	65 (89%)	68 (88%)	0.888
Current smoker, n (%)	26 (36%)	27 (35%)	0.853
Current alcohol drinker, n (%)	33 (46%)	32 (43%)	0.643
Pharmacological treatment, n (%)	35 (48%)	59 (77%)	**<0.001**
Diuretics, n (%)	6 (8%)	12 (16%)	0.165
Anticoagulants, n (%)	5 (7%)	11 (14%)	0.140
Antacids, n (%)	9 (12%)	15 (19%)	0.232
Hypolipidemics, n (%)	10 (14%)	28 (36%)	**0.001**
Beta-blockers, n (%)	4 (5%)	6 (8%)	0.570
Calcium antagonists, n (%)	5 (7%)	12 (16%)	0.092
ACEi, n (%)	14 (19%)	26 (34%)	**0.043**
Antiarrhythmics, n (%)	0 (0%)	2 (3%)	0.166
Insulin, n (%)	1 (1%)	2 (3%)	0.591
Oral antidiabetics, n (%)	2 (3%)	9 (12%)	**0.036**

Mean (SD) or n (%) as appropriate. OSA: obstructive sleep apnea; AHI: apnea-hypopnea index; SaO_2_: oxygen saturation; ACEi: angiotensin-converting enzyme inhibitors

* Independent samples Student’s t test or chi-square test as appropriate

**Table 2:** Hormonal and biochemical profiles of OSA patients according to erectile dysfunction (ED) presence.

	non-ED n = 73	ED n = 77	p-value*
Testosterone, nmol/L	11.3 (3.7)	11.5 (4.0)	0.840
SHBG, nmol/L	29 (23–36)	33 (27–44)	0.058
Free testosterone, %	37 (29–46)	33 (27–39)	0.066
Prolactin, μg/ml	8.6 (4.2)	8.1 (3.3)	0.400
LH, IU/l	4.0 (2.4)	3.6 (1.7)	0.243
FSH, IU/l	6.4 (5.3)	5.9 (3.3)	0.501
Leukocytes, 10^9^ /L	7.6 (1.9)	7.6 (1.7)	0.950
Hemoglobin, g /dL	16 (1)	16 (1)	0.257
Platelets, 10^9^ /L	225 (58)	226 (41)	0.886
Glucose, mg /dL	94 (13)	107 (40)	**0.024**
Total cholesterol, mg /dL	209 (32)	198 (35)	0.059
HDL cholesterol, mg /dL	49 (11)	47 (8.8)	0.239
LDL cholesterol, mg /dl	135 (30)	125 (33)	0.075
Triglycerides, mg /dL	153 (84)	153 (75)	0.980
Proteins, g /dL	7 (0.3)	7 (0.3)	0.760
CRP, mg /L	3.8 (3.3)	5.1 (7)	0.185

Mean (SD) or median (IQR) as appropriate. OSA: obstructive sleep apnea; SHBG: sex hormone binding globulin; LH: luteinizing hormone; FSH: follicle-stimulating hormone; HDL: high-density lipoprotein; LDL: low-density lipoprotein; CRP: C-reactive protein

* Independent samples Student’s t test or Mann-Whitney U test as appropriate

**Table 3:** Erectile function and self-esteem and relationship scores of OSA patients according to erectile dysfunction (ED) presence.

	non-ED n = 73	ED n = 77	p-value*
**IIEF15**			
Erectile function	29 (27–30)	18 (6–22)	**<0.001**
Orgasmic function	10 (9–10)	8 (3–10)	**<0.001**
Sexual desire	7 (6–9)	6 (4–7)	**<0.001**
Sexual satisfaction	12 (11–13)	8 (4–11)	**<0.001**
Overall satisfaction	8 (8–9)	6 (4–8)	**<0.001**
**SEAR**			
Sexual relations	81 (72–88)	63 (50–72)	**<0.001**
Self-confidence	75 (67–83)	63 (46–79)	**<0.001**
Self-esteem	75 (59–81)	56 (44–75)	**0.002**
Relations	88 (75–100)	75 (50–100)	**0.001**
Total score	78 (71–86)	62 (50–77)	**<0.001**

Median (IQR). OSA: obstructive sleep apnea; IIEF15: International Index Erectile Function test; SEAR: Self-esteem and Relationship test

* Mann-Whitney U test

After randomization 40 patients were treated with CPAP for 3 months with a median (IQR) CPAP use of 5.3 (3.2–6.0) h/night. Eight non-CPAP and 10 CPAP subjects had no IIEF15 measurements at the end of the follow-up and were excluded from the primary outcome analyses. A baseline comparison between study arms revealed no significant differences except for AHI (mean (SD) events·h^−1^: CPAP patients 55.8 (20.6); non-CPAP patients 45.3 (19.2); p = 0.037), although no statistically significant differences were found in the proportion of severe (AHI > 30 events·h^−1^) OSA patients. Significant increases in erectile function (mean (SD) change: +4.6 (7.9); p = 0.002), sexual satisfaction (mean (SD) change: +2.1 (4.3); p = 0.003) and overall satisfaction (mean (SD) change: +1 (2.2); p = 0.035) were found (Table 4). Between-arm differences in sexual satisfaction changes were statistically significant (p = 0.027), while differences in erectile function changes were borderline non-significant (p = 0.060). Regardless of statistical significance, dose-response relationships could be seen for Erectile function and Sexual satisfaction when considering 3 CPAP compliance categories: non-users, user below median (5.3 h/night) and users above median (Table A in S1 Additional Results). CPAP treatment did not have any relevant impacts on the psychological, hormonal or biochemical profiles of ED patients (Tables 5 and 6). Finally, ordered logistic regression adjusting for age, WHR, and use of hypolipidemics, that were the most differing variables between ED and non-ED patients, showed very similar results (data not shown).

**Table 4:** Effect of CPAP treatment on the erectile function of OSA patients with ED.

IIEF15		non-CPAP (n = 27)				CPAP (n = 30)			p-value†
	Baseline	Follow-up	Change	p-value*	Baseline	Follow-up	Change	p-value*	
Erectile function	15.7 (9.1)	17.9 (9.1)	+2.1 (5.9)	**0.024**	15.2 (7.9)	19.8 (9.0)	+4.6 (7.9)	**0.002**	0.060
Orgasmic function	6.2 (4.0)	6.5 (3.9)	+0.3 (3.8)	0.911	6.8 (3.4)	7.3 (3.5)	+0.4 (3.6)	0.213	0.413
Sexual desire	5.3 (2.4)	5.9 (2.2)	+0.6 (1.5)	0.057	5.6 (1.4)	6.2 (2.0)	+0.6 (1.7)	0.102	0.968
Sexual satisfaction	7.6 (4.5)	7.9 (4.9)	+0.3 (4.1)	0.238	7.2 (4.1)	9.4 (4.7)	+2.1 (4.3)	**0.003**	**0.027**
Overall satisfaction	5.7 (2.5)	6.0 (2.4)	+0.4 (1.6)	0.259	6.3 (2.4)	7.3 (2.3)	+1.0 (2.2)	**0.035**	0.348

Mean (SD). Intention to treat analysis. Of the 75 randomized patients, 8 non-CPAP and 10 CPAP subjects had no IIEF15 measurements at the end of the follow-up and were excluded from these analyses. OSA: obstructive sleep apnea; IIEF15: International Index Erectile Function test

* Wilcoxon’s matched-pairs signed-ranks test comparing baseline and final measures

† Wilcoxon’s rank-sum test comparing the changes from baseline to the end of follow-up between non-CPAP and CPAP patients

**Table 5:** Effect of CPAP treatment on the self-esteem and relationship scores of OSA patients with ED.

SEAR		non-CPAP (n = 27)				CPAP (n = 30)			p-value†
	Baseline	Follow-up	Change	p-value*	Baseline	Follow-up	Change	p-value*	
Sexual relations	59 (19)	62 (23)	+3 (18)	0.370	59 (24)	64 (21)	+5 (21)	0.138	0.602
Self-confidence	62 (23)	66 (17)	+4 (21)	0.087	63 (21)	67 (22)	+4 (17)	0.083	0.811
Self-esteem	60 (26)	62 (20)	+2(23)	0.334	58 (22)	64 (23)	+6 (18)	0.054	0.483
Relations	69 (25)	76 (16)	+7 (27)	0.193	71 (25)	71 (25)	-0 (21)	0.908	0.333
Total score	61 (19)	64 (19)	+3 (17)	0.178	60 (20)	64 (18)	+4 (18)	0.147	0.810

Mean (SD). Intention to treat analysis. Of the 75 randomized patients, 8 non-CPAP and 10 CPAP subjects had no SEAR measurements at the end of the follow-up and were excluded from these analyses. OSA: obstructive sleep apnea; SEAR: Self-esteem and Relationship test

* Wilcoxon’s matched-pairs signed-ranks test comparing baseline and final measures

† Wilcoxon’s rank-sum test comparing the changes from baseline to the end of follow-up between non-CPAP and CPAP patients

**Table 6:** Effect of CPAP treatment on the hormonal and biochemical profiles of OSA patients with ED.

		non-CPAP (n = 28)				CPAP (n = 32)			p-value†
	Baseline	Follow-up	Change	p-value*	Baseline	Follow-up	Change	p-value*	
Testosterone, nmol/L	11 (4)	12 (3)	+0.3 (3.0)	0.358	12 (4)	11 (3)	-0.6 (2.4)	0.291	0.131
SHBG, nmol/L	36 (14)	37 (16)	+1.3 (8.0)	0.991	37 (15)	34 (16)	-2.3 (7.0)	0.077	0.119
Free testosterone, %	35 (15)	35 (13)	+0.7 (10.7)	0.545	34 (10)	35 (10)	+0.9 (6.8)	0.395	0.801
Prolactin, μg/ml	8.1 (4.1)	7.2 (2.7)	-0.9 (3.7)	0.112	7.9 (2.3)	7.5 (2.2)	-0.4 (1.9)	0.405	0.340
LH, IU/l	3.5 (1.6)	3.8 (1.9)	+0.3 (1.3)	0.144	3.7 (1.9)	3.4 (1.6)	-0.3 (1.4)	0.178	**0.042**
FSH, IU/l	5.4 (2.7)	5.6 (2.7)	+0.2 (0.8)	0.160	6.5 (3.9)	6.5 (3.7)	+0.0 (0.8)	0.645	0.248
Leukocytes, 10^9^ /L	7.7 (1.8)	7.5 (1.9)	-0.2 (2.1)	0.116	7.5 (1.6)	6.9 (1.4)	-0.6 (1.2)	**0.014**	0.830
Haemoglobin, g /dL	16 (0.8)	16 (0.9)	+0.1 (0.7)	0.608	16 (1.2)	15 (1.4)	-0.3 (0.6)	**0.004**	**0.033**
Platelets, 10^9^ /L	235 (40)	226 (45)	-8.7 (20)	**0.021**	219 (39)	214 (42)	-4.7 (22)	0.155	0.430
Glucose, mg /dL	100 (27)	100 (23)	+0.2 (12)	0.888	106 (37)	105 (32)	-0.5 (19)	0.469	0.693
Total cholesterol, mg /dL	195 (28)	201 (38)	+6.6 (31)	0.275	197 (39)	191 (38)	-6.4 (32)	0.376	0.217
HDL cholesterol, mg /dL	48 (7.8)	48 (8.0)	-0.2 (5.4)	0.862	46 (9.2)	47 (9.2)	+1.4 (7.1)	0.474	0.641
LDL cholesterol, mg /dl	122 (28)	123 (37)	+1 (31)	0.754	123 (37)	118 (33)	-5.4 (30)	0.469	0.481
Triglycerides, mg /dL	159 (89)	194 (216)	+35 (222)	0.729	142 (61)	151 (81)	+9.8 (69)	0.472	0.834
Proteins, g /dL	7.1 (0.3)	7.2 (0.3)	+0.0 (0.3)	0.517	7.0 (0.3)	7.0 (0.3)	+0.0 (0.2)	0.347	0.739
CRP, mg /L	3.9 (2.9)	3.2 (2.0)	-0.7 (2.0)	0.181	5.5 (8.7)	3.1 (2.0)	-2.4 (8.3)	0.095	0.809

Mean (SD). Intention to treat analysis. Of the 75 randomized patients, 7 non-CPAP and 8 CPAP subjects had no hormonal and biochemical measurements at the end of the follow-up and were excluded from this analysis. OSA: obstructive sleep apnea; HDL: high-density lipoprotein; LDL: low-density lipoprotein; CRP: C-reactive protein

* Wilcoxon’s matched-pairs signed-ranks test comparing baseline and final measures

† Wilcoxon’s rank-sum test comparing the changes from baseline to the end of follow-up between non-CPAP and CPAP patients

## Discussion

In this double-purpose study, we determined an ED prevalence of 51% among newly diagnosed OSA patients. Patients with ED were older, had a greater WHR, were more frequently undergoing pharmacological treatment and had higher glucose levels than patients without ED. Although after 3 months of CPAP treatment patients improved on erectile function, sexual satisfaction and overall satisfaction, only changes in sexual satisfaction were significantly different than changes in the control group. Changes in erectile function among study arms were on the borderline of statistical significance, likely due to difficulties in achieving the planned sample size. Finally, CPAP treatment did not have any relevant impacts on the psychological, hormonal or biochemical profiles of OSA patients with ED.

The prevalence of ED among newly diagnosed OSA patients in our study was 51%. This prevalence was lower than those reported by Budweiser (69%) [7] and Giner (69%) [20]. These differences could be explained by the inclusion in previous studies of patients with known ED risk factors, such as uncontrolled or advanced chronic conditions, drug addiction, or age greater than 70 years. As expected and according to the previous literature, we found a higher ED prevalence among the oldest patients. Similarly, ED patients more commonly used drugs, specifically hypolipidemic, angiotensin-converting enzyme inhibitors and oral antidiabetics. Differences in WHR, but not in weight or BMI, were found, in agreement with studies showing that central obesity is an independent ED risk factor [21,22]. The absence of differences in the biochemical profiles of OSA patients with and without ED validated the decision to exclude all patients with uncontrolled cardiovascular risk factors for ED. Regarding hormonal profiles, ED patients had slightly higher sHBG and lower free testosterone levels than non-ED patients. These differences were most likely explained by age differences because plasma sHBG concentration increases and total testosterone decreases with age, leaving less free testosterone available [23]. Finally, although some studies have suggested that OSA could be related to dysfunction in the hypothalamic-pituitary axis [24], no differences in prolactin, LH or FSH levels were found, probably due to hormonally active testosterone levels being within normal ranges and gonadotrophins therefore not being activated.

Although improvements in erectile function, sexual satisfaction and overall satisfaction were found after 3 months of CPAP treatment, only the change in sexual satisfaction was significantly different between study arms while changes in erectile function were on the borderline of statistical significance. After CPAP treatment, the IIEF15 erectile function score was 19.8, which was almost the same as that reported by Taskin in 2010, after 1 month of CPAP treatment [25], and it did not reach the 25 points that is the threshold for normal values. Other studies to date have reported different degrees of ED improvement after CPAP treatment [7,26–35]. It is noteworthy that the most similar previous study in terms of design, an RCT assessing the effect of 3 months of CPAP / sham CPAP on IIEF15 domains by Melehan et al in 2018, showed a similar increase in sexual satisfaction and non-significant changes on erectile function [27]. Both RCT [29,31] and observational [28,30,35] studies assessing the short-term (1 to 3 months) effect of CPAP on ED as measured by IIEF5, that cannot assess the different domains of ED, have reported moderately positive results, with the exception of Shin et al that found no effect of CPAP treatment [32]. On the other hand, the only study assessing long-term effects of CPAP treatment on ED, an observational study by Budweiser et al using IIEF15 as primary end point, showed that CPAP treatment increased overall IIEF15 score and was able to halt the decrease in sexual function seen among controls [34]. Treatment with sildenafil has proved to be more effective than CPAP [31,36,37] in improving ED symptoms in OSA patients, although the combination of CPAP and sildenafil has been reported to provide some additional improvements [38]. In contrast, the effect of CPAP on sexual satisfaction in our RCT suggested an increase in the patient’s perception of well-being. Moreover, the positive effects of CPAP treatment, both in terms of snoring reduction and sleep quality, could have a positive impact on the partner’s sleep quality and overall intimacy levels. This is a novel and relevant result because previous studies assessing ED with the abbreviated version of the IIEF could not assess ED dimensions beyond erectile function [25].

The relationship between OSA and ED is well established [5–7]. Testosterone, which peaks during the Rapid Eyes Movements (REM) phase, is usually lower in OSA patients due to sleep fragmentation [39] and, along with repeated hypoxia episodes, could lead to a decrease in protective nocturnal erectile activity [40]. However, the current study did not find significant differences in total or free testosterone levels between non-ED and ED groups. Adequate compliance with CPAP treatment could potentially reverse or stop the detrimental effects of OSA on ED. However, in our RCT, patients receiving CPAP treatment did not perform better than patients in the control arm. This finding could be partially explained by the characteristics of the included patients, who consulted the hospital because of sleep problems and not ED-related symptoms. In such patients, questions regarding sexual function (that until that time had not caused the patient any reason for consultation) could increase awareness of the situation and promote attitude changes toward any ED-related problems.

Our results emphasize the importance of OSA screening in patients undergoing consultations for ED, and vice versa, given the high prevalence of ED among OSA patients even after excluding patients with many known ED risk factors. Accordingly, measurements such as WHR could be used for the identification of OSA patients at higher risk for ED. In contrast, CPAP treatment showed limited success in ameliorating ED, suggesting that CPAP treatment should probably be combined with other therapeutic options, such as weight reduction or sildenafil, depending on ED severity, to fully overcome ED. In any case, patients’ awareness of both OSA and ED could potentially be useful for achieving better treatment adherence.

The current study had several strengths: (i) the inclusion of newly diagnosed OSA patients, all of whom had undergone complete polysomnography; (ii) the exclusion of patients with many well-known ED risk factors, such as uncontrolled or advanced chronic conditions, drug addiction, and age greater than 70 years, in an effort to study OSA-related ED without additional confounding variables; (iii) comprehensive determination of the profiles of OSA patients with and without ED, including a broad range of sociodemographic, anthropometric, lifestyle, hormonal, biochemical, clinical and pharmacological variables; and (iv) the use of an RCT design to assess the potential effects of CPAP treatment on ED. In contrast, several limitations should be acknowledged: (i) Although having a randomization goal of 126 ED patients, the study was stopped at 75. The main reasons for not reaching the recruitment goal were, first, the lower-than-expected prevalence of ED coupled with tight inclusion criteria, causing a very slow recruitment pace (a planned 2-year study turned out having a 3.5 years recruitment phase); second, the financial constrains caused by the extended recruitment phase; and third, the need to avoid biases caused by changes in the personnel assessing the patients. This fact diminished the statistical power of the RCT, most likely precluding statistical significance of the differences in changes in erectile function between CPAP and non-CPAP patients. Nevertheless, it is unlikely that increasing the sample size would alter the null results in the psychosocial aspects or hormonal and biochemical profiles investigated; (ii) there were no repeated measures of hormonal profile. This could cause some degree of measurement error specially in hormones such as testosterone that are subject to short-term fluctuations; (iii) the RCT phase of this study had a non-blind design. Although the use of sham CPAP could allow for a blind design, this option was rejected both to avoid unnecessary discomfort to patients and to avoid neglecting the potential negative implications of using a medical device while sleeping in relation to sexual behavior and/or performance; and, (iv) the Ethics Committee approving the study precluded the inclusion of sleepy patients (ESS score >10) and limited the follow-up time to 3 months, as not providing CPAP treatment to sleepy patients or delaying CPAP treatment initiation beyond 3 months in non-sleepy patients is considered unethical. This implied that the current study could not provide insight into the long-term effects of CPAP treatment on ED. Moreover, this excluded from the study a part of OSA patients (usually with a more severe disease). Nevertheless, the inclusion of sleepy patients would most provably favor the authors hypotheses, enhancing the bonds between OSA and ED, and potentially magnifying the beneficial effects of CPAP on ED.

To conclude, we found a 51% prevalence of ED among newly diagnosed OSA patients, even when excluding patients with many known ED risk factors. Age, WHR and pharmacological treatment were identified as the main differences between OSA patients with and without ED. Finally, 3 months of CPAP treatment significantly increased sexual satisfaction but had no impact on the psychological, hormonal or biochemical profiles of patients. Overall, this study confirms the close relationship between OSA and ED, suggesting the potential usefulness of ED screening in OSA patients, but could not determine conclusively whether CPAP is an effective stand-alone ED treatment despite positive results on sexual satisfaction.

## Supporting information

S1 Consort Checklist (DOC)

S1 Questionnaires Main questionnaires used in the study. (PDF)

S1 Additional Results (DOCX)

S1 Protocol Spanish protocol. (PDF)

S2 Protocol English protocol. (PDF)
