# Supplementary material for: Erectile dysfunction in obstructive sleep apnea patients: A randomized trial on the effects of Continuous Positive Airway Pressure (CPAP)
Source: PLoS One. 2018 Aug 8;13(8):e0201930. doi: 10.1371/journal.pone.0201930 (PMC6082539; doi:10.1371/journal.pone.0201930)
Supplement: S1 Questionnaires — (PDF) [file pone.0201930.s002.pdf]

INDICE INTERNACIONAL DE FUNCION ERECTIL - IIEF -

Iniciales del paciente ____________ Nº identificación ____________ Fecha ____________

¿Es usted sexualmente activo (definitivo como actividad sexual con pareja o estimulación manual)?

☐ **SI** ☐ **NO**

En caso afirmativo, por favor cumplimente el cuestionario relativo a su actividad sexual. (en cada pregunta, marque sólo una casilla).

	SIN ACTIVIDAD SEXUAL	CASI NUNCA ó NUNCA	MENOS DE LA MITAD DE LAS VECES	LA MITAD DE LAS VECES	MAS DE LA MITAD DE LAS VECES	CASI SIEMPRE ó SIEMPRE
1. Durante las últimas 4 semanas, ¿con qué frecuencia logró una erección durante la actividad sexual[B]?	0	1	2	3	4	5
2. Durante las últimas 4 semanas, cuando tuvo erecciones con la estimulación sexual[D], ¿con qué frecuencia fue suficiente la rigidez para la penetración?	0	1	2	3	4	5
3. Durante las últimas 4 semanas, al intentar una relación sexual[A], ¿con qué frecuencia logró penetrar a su pareja?	0	1	2	3	4	5
4. Durante las últimas 4 semanas, durante la relación sexual[A], ¿con qué frecuencia logró mantener la erección después de la penetración?	0	1	2	3	4	5

	SIN ACTIVIDAD SEXUAL	EXTREMADAMENTE DIFICIL	MUY DIFICIL	DIFICIL	ALGO DIFICIL	SIN DIFICULTAD
5. Durante las últimas 4 semanas, durante la relación sexual[A], ¿Cuál fue el grado de dificultad para mantener la erección hasta completar la relación sexual?	0	1	2	3	4	5

	NINGUNA	1-2 VECES	3-4 VECES	5-6 VECES	7-10 VECES	11 ó MAS
6. Durante las últimas 4 semanas, ¿cuántas veces intentó una relación sexual[A]?	0	1	2	3	4	5

	SIN ACTIVIDAD SEXUAL	CASI NUNCA ó NUNCA	MENOS DE LA MITAD DE LAS VECES	LA MITAD DE LAS VECES	MAS DE LA MITAD DE LAS VECES	CASI SIEMPRE ó SIEMPRE
7. Durante las últimas 4 semanas, cuando intentó una relación sexual[A], ¿con qué frecuencia resultó satisfactoria para usted?	0	1	2	3	4	5

A = Acto sexual;
Se define como la penetración de la pareja.

B = Actividad sexual;
Incluye el acto sexual, caricias juegos anteriores al acto y la masturbación.

C = Eyacular;
Se define como la expulsión de semen del pene (o la **sensación** de hacerlo).

D = Estimulación sexual;
Incluye situaciones como juegos amorosos con una pareja o mirar fotos eróticas, etc.

INDICE INTERNACIONAL DE FUNCION ERECTIL - IIEF - (cont.)

	NO REALICE EL ACTO	NO DISFRUTE NADA	NO DISFRUTE MUCHO	DISFRUTE ALGO	DISFRUTE BASTANTE	DISFRUTE MUCHO
8. Durante las últimas 4 semanas, ¿cuánto ha disfrutado de la relación sexual[A]?	0	1	2	3	4	5

	SIN ACTIVIDAD SEXUAL	CASI NUNCA ó NUNCA	MENOS DE LA MITAD DE LAS VECES	LA MITAD DE LAS VECES	MAS DE LA MITAD DE LAS VECES	CASI SIEMPRE ó SIEMPRE
9. Durante las últimas 4 semanas, durante la estimulación o la relación sexual[A], ¿con qué frecuencia eyaculó[C]?	0	1	2	3	4	5
10. Durante las últimas 4 semanas, durante la estimulación[C] o la relación sexual[A], ¿con qué frecuencia tuvo una sensación de orgasmo[C] (con o sin eyaculación)?	0	1	2	3	4	5

Las siguientes dos preguntas se refieren al deseo sexual, definido como una sensación que puede ser un deseo de tener una experiencia sexual (por ejemplo, masturbación o relación sexual), un pensamiento sobre una relación sexual o un sentimiento de frustración por no tener una relación sexual.

	CASI NUNCA ó NUNCA	EN ALGUNOS MOMENTOS	BUENA PARTE DEL TIEMPO	LA MAYOR PARTE DEL TIEMPO	CASI SIEMPRE ó SIEMPRE
11. Durante las últimas 4 semanas, ¿con qué frecuencia ha sentido un deseo sexual?	1	2	3	4	5

	MUY BAJO ó NULO	BAJO	MODERADO	ALTO	MUY ALTO
12. Durante las últimas 4 semanas, ¿cómo calificaría su nivel de deseo sexual?	1	2	3	4	5

	MUY INSATISFECHO	BASTANTE INSATISFECHO	NI SATISFECHO NI INSATISFECHO	BASTANTE SATISFECHO	MUY SATISFECHO
13. Durante las últimas 4 semanas, ¿cuál ha sido el grado de satisfacción con su vida sexual en general?	1	2	3	4	5
14. Durante las últimas 4 semanas, ¿cuál ha sido el grado de satisfacción con la relación sexual con su pareja?	1	2	3	4	5

	MUY BAJO ó NULO	BAJO	MODERADO	ALTO	MUY ALTO
15. Durante las últimas 4 semanas, ¿cómo calificaría la confianza que tiene en poder lograr y mantener una erección?	1	2	3	4	5

A = Acto sexual;
Se define como la penetración de la pareja.

B = Actividad sexual;
Incluye el acto sexual, caricias juegos anteriores al acto y la masturbación.

C = Eyacular;
Se define como la expulsión de semen del pene (o la sensación de hacerlo).

D = Estimulación sexual;
Incluye situaciones como juegos amorosos con una pareja o mirar fotos eróticas, etc.

Cuestionario de Autoestima y Relaciones (Versión Española del cuestionario SEAR)

Por favor, al responder a las siguientes cuestiones, piense en las 4 últimas semanas.
Escoja una sola respuesta para cada cuestión y señale la casilla con una X.

	**Casi siempre o siempre**	**La mayoría de las veces** *(mucho más de la mitad de las veces)*	**Algunas veces** *(aproximadamente la mitad de las veces)*	**Pocas veces** *(mucho menos de la mitad de las veces)*	**Casi nunca o nunca**
1.- Me he sentido tranquilo al pensar en empezar cada relación sexual con mi pareja	5	4	3	2	1
2.- Me he sentido seguro de que mi erección duraría lo suficiente durante la relación sexual	5	4	3	2	1
3.- Me he sentido satisfecho de cómo he funcionado sexualmente	5	4	3	2	1
4.- He sentido que podría tener relaciones sexuales cuando surgieran de forma espontánea	5	4	3	2	1
5.- Me he sentido predispuesto a empezar yo las relaciones sexuales	5	4	3	2	1
6.- Me he sentido seguro de funcionar sexualmente	5	4	3	2	1
7.- Me he sentido satisfecho con nuestra vida sexual	5	4	3	2	1
8.- Mi pareja se ha sentido descontenta con la calidad de nuestras relaciones sexuales	5	4	3	2	1
9.- Me he sentido bien conmigo mismo	5	4	3	2	1
10.- Me he sentido como un hombre completo	5	4	3	2	1
11.- Estuve propenso a sentir que soy un fracasado	5	4	3	2	1
12.- Me he sentido seguro de mi mismo	5	4	3	2	1
13.- Mi pareja se ha sentido satisfecha con nuestra relación en general	5	4	3	2	1
14.- Me he sentido satisfecho con nuestra relación en general	5	4	3	2	1

	**Muy satisfecho**	**Bastante satisfecho**	**Ni insatisfecho ni satisfecho**	**Bastante insatisfecho**	**Muy insatisfecho**
¿Cómo se sentiría si tuviera que pasar el resto de su vida con su grado de erección actual?	5	4	3	2	1

Gestió de
Serveis Sanitaris

Institut Català de la Salut
Lleida
Hospital Universitari Arnau de Vilanova

ESCALA DE SOMNOLENCIA DE EPWORTH

INSTRUCCIONES

¿Llega a veces a dormitar o a dormirse, y no sólo a sentirse cansado, en las siguientes situaciones?
Esta pregunta se refiere a su vida habitual durante los últimos meses. Incluso si usted no se ha encontrado recientemente en una de las siguientes situaciones, trate de imaginar cómo le podría haber afectado.
Elija de la siguiente escala la puntuación más apropiada para cada situación.

0 = no me dormiría nunca
1 = ligera posibilidad de dormirme
2 = mediana posibilidad de dormirme
3 = muchas posibilidades de dormirme

SITUACIÓN	PUNTUACIÓN
Sentado leyendo	....
Mirando la televisión	....
Sentado, inactivo en un lugar público (teatro, reunión)	....
Como pasajero en un coche circulando durante una hora sin pararse	....
Tumbado por la tarde para descansar, cuando las circunstancias me lo permite	....
Sentado hablando con alguien	....
Sentado tranquilamente después de una comida sin alcohol	....
En el coche parado algunos minutos en un atasco	....
PUNTUACIÓN GLOBAL	**...........**

Gracias por su colaboración

Fecha: __/__/__ Firma:
