# Supplementary material for: Erectile dysfunction in obstructive sleep apnea patients: A randomized trial on the effects of Continuous Positive Airway Pressure (CPAP)
Source: PLoS One. 2018 Aug 8;13(8):e0201930. doi: 10.1371/journal.pone.0201930 (PMC6082539; doi:10.1371/journal.pone.0201930)
Supplement: S1 Additional Results — (DOCX) [file pone.0201930.s003.docx]

| **Table A. Changes in IIEF scores from baseline to end of follow-up according to CPAP compliance categories.** | | | | |
| --- | --- | --- | --- | --- |
| **IIEF** | **CPAP compliance** | | |  |
|  | Non-users (n=28) | ≤ 5.3 h/night (n=17) | > 5.3 h/night (n=14) | p-trend* |
| Erectile function | +2.3 (5.9) | +3.9 (9.3) | +5.6 (5.7) | 0.139 |
| Orgasmic Function | +0.3 (3.7) | +0.8 (4.7) | +0.2 (1.8) | 0.994 |
| Sexual desire | +0.6 (1.5) | +0.6 (1.5) | +0.5 (2.0) | 0.847 |
| Sexual satisfaction | +0.4 (4.1) | +1.7 (5.1) | +2.4 (3.0) | 0.142 |
| Overall Satisfaction | +0.4 (1.6) | +1.1 (2.6) | +1.0 (1.9) | 0.327 |
| Mean (SD). The cutoff value (5.3) corresponds to the median CPAP compliance. IIEF: International Index Erectile Function test. CPAP: Continuous Positive Airway Pressure treatment. * P-values of linear regression models assessing the changes in IIEF dimensions’ scores from baseline to end of follow-up using the categorized CPAP compliance variable (non-users; ≤ 5.3 h/night; and, > 5.3 h/night) as a continuous variable. | | | | |
