# Supplementary material for: Erectile dysfunction in obstructive sleep apnea patients: A randomized trial on the effects of Continuous Positive Airway Pressure (CPAP)
Source: PLoS One. 2018 Aug 8;13(8):e0201930. doi: 10.1371/journal.pone.0201930 (PMC6082539; doi:10.1371/journal.pone.0201930)
Supplement: S1 Protocol — (PDF) [file pone.0201930.s004.pdf]

MEMORIA DE SOLICITUD DEL PROYECTO DE INVESTIGACIÓN

TITULO:
Impacto del Síndrome de Apnea Obstructiva del Sueño en la Disfunción Eréctil

Investigador principal: Mercè Pascual Queralt

RESUMEN (Objetivos y metodología del proyecto): (Máximo 250 palabras)

Introducción: El síndrome de apneas obstructivas del sueño (SAOS) es un proceso frecuente que afecta al 2-4% de la población general, provoca un aumento de la actividad simpática, cambios de la presión arterial sistémica, y se asocia con enfermedad cardiovascular. Dichas alteraciones pueden tener un impacto en los mecanismos de la erección. Hasta la fecha actual, los estudios que relacionan disfunción eréctil con Síndrome de Apnea Obstructiva del Sueño (SAOS) son de tipo epidemiológico.

Objetivos: Determinar el impacto del SAOS en la disfunción eréctil tanto desde el punto de vista orgánico (trastornos cardiovasculares, alteración de los niveles hormonales, alteraciones loco-regionales) como psicológico (evaluación psicológica del paciente SAOS que presenta disfunción eréctil). Para ello vamos a determinar el perfil de síntesis de marcadores biológicos relacionados con disfunción endotelial y trastorno cardiovascular que resultan alterados como consecuencia del SAOS y su relación con la presencia de disfunción eréctil. Además, se evaluará el perfil de secreción de hormonas sexuales con el objetivo de determinar si existe un patrón alterado en pacientes SAOS con disfunción eréctil, respecto a pacientes SAOS sin disfunción eréctil. Asimismo, uno de los objetivos del presente estudio será evaluar el impacto del tratamiento con CPAP (3 meses) en el perfil de expresión de los marcadores analizados relacionados con disfunción endotelial.

Metodología: Estudio prospectivo multicéntrico en el que participarán el Hospital Arnau de Vilanova y Hospital Santa María de Lleida. Se incluirán pacientes que acudan a la Unidad del Sueño para el diagnóstico de SAOS mediante polisomnografía convencional. Además, se les realizará: Test Epworth, Pulsioximetría, perfil bioquímico básico, análisis de marcadores biológicos (disfunción endotelial y trastorno cardiovascular, y hormonas sexuales), test IIEF (International Index Erectile Function) y Cuestionario SEAR. Todos aquellos con diagnóstico de SAOS, tras recibir el tratamiento con CPAP durante 3 meses volverán a ser estudiados, con el fin de evaluar el impacto del tratamiento con CPAP en los marcadores relacionados con disfunción eréctil en el paciente con SAOS.

MEMORIA DE SOLICITUD DEL PROYECTO DE INVESTIGACIÓN

Investigador principal: Mercè Pascual Queralt

Antecedentes y estado actual del tema (Citar las referencias incluidas en el apartado siguiente) (Máximo 3 páginas)

El síndrome de apneas - hipopneas del sueño (SAHS) se caracteriza por la presencia de pausas respiratorias totales (apneas) o parciales (hipopneas), debidas al colapso de la vía aérea superior durante el sueño. Es un proceso frecuente que afecta al 2-4% de la población general y clínicamente se caracteriza por la presencia de ronquido, somnolencia diurna y deterioro de la calidad de vida (1). El índice apnea-hipopnea (AHI) representa el número de apneas o hipopneas por hora de sueño y es la forma habitual de definir la enfermedad. Su tratamiento consiste en cambios en el estilo de vida como pérdida de peso y la aplicación de presión positiva continua por vía nasal (CPAP) durante el sueño. La CPAP es un tratamiento efectivo ya que corrige los eventos respiratorios obstructivos, mejora la sintomatología y la calidad de vida de los pacientes afectos de Síndrome de apnea obstructiva del sueño (SAOS). Cada episodio de apnea se acompaña de un descenso en la saturación arterial de oxígeno, que rápidamente se normaliza con la siguiente ventilación (hipoxia-reoxigenación), cambios bruscos en la presión intrapleural. Se ha demostrado que a medida que progresa la noche se produce un aumento en el número y duración de las apneas, aumentando el grado de desaturación de oxígeno y los niveles de presión intratorácica generados en cada apnea (1,2,3). Cada evento apneico finaliza con un arousal (despertar electroencefalográfico). Los episodios de hipoxia intermitente y los arousals provocan un aumento de la actividad simpática y cambios repentinos de la presión arterial sistémica, que contribuyen al desarrollo de hipertrofia miocárdica, arritmias cardiacas y muerte. Actualmente, el SAHS se considera como la principal causa de hipertensión arterial secundaria, se asocia a enfermedad cardiovascular y constituye un problema socio-sanitario relevante.

Además, los episodios repetidos de hipoxia-reoxigenación, y el aumento de la actividad simpática pueden activar diferentes vías patogénicas que favorecen la aterogénesis como son: stress oxidativo, disfunción endotelial, hipercoagulabilidad y alteraciones metabólicas como la resistencia a la insulina. Estas alteraciones fisiológicas explicarían el mayor riesgo de desarrollar enfermedad cardiovascular en los pacientes con SAHS (4-7).

Entre las afectaciones del sistema vascular se pueden alterar los mecanismos de la erección, un fenómeno neurovascular bajo control hormonal que incluye dilatación arterial, relajación de la musculatura lisa y activación del sistema veno-oclusivo de los cuerpos cavernosos (8). De forma fisiológica se producen erecciones espontáneas durante el sueño. Su finalidad es la de oxigenar el tejido eréctil para mantener su vitalidad. Se sabe que ante una erección completa es cuando el tejido cavernoso tiene una mayor oxigenación, pero hay estudios que sugieren que una baja presión intracavernosa, o sea erecciones parciales, también permiten una suficiente oxigenación del tejido eréctil y evitan el daño producido por la hipoxia (9).

La disfunción eréctil es la imposibilidad de alcanzar y mantener una erección suficiente para tener una relación sexual satisfactoria durante almenos tres meses, salvo en los casos de trauma o cirugía (10). Aunque sea un trastorno benigno, afecta también la salud psicosocial del paciente y tiene un impacto importante en la calidad de vida tanto de los pacientes como su pareja y entorno familiar (11). A nivel mundial se han realizado varios estudios epidemiológicos para determinar la prevalencia de disfunción eréctil, que ha variado entre 12% - 71% en función de la metodología, el grupo de estudio, el tamaño muestral y la definición utilizada de disfunción eréctil. También se han realizado estudios de relación entre disfunción eréctil y enfermedades como hipertensión, enfermedad cardiovascular, dislipemia, diabetes y depresión (12). Existen varios factores que pueden explicar el origen de la disfunción eréctil. Factores de riesgo cardiovascular, alteraciones neuroendocrinas, factores de riesgo psicológico y alteraciones orgánicas locales secundarios a cirugías regionales, malformaciones congénitas o malformaciones adquiridas.

Hasta la fecha actual, los estudios que relacionan disfunción eréctil con SAOS son de tipo epidemiológico, basados en estudios descriptivos transversales, que incluyen muchas variables y donde se encuentran múltiples factores de confusión. Al mismo tiempo, muchos enfermos con SAOS son roncadores crónicos, cosa que puede conllevar un peor descanso tanto del paciente como de la propia pareja y un menor rendimiento en la actividad sexual. Ningún estudio previo ha realizado una evaluación de los marcadores biológicos (marcadores cardiovasculares y hormonas sexuales) que resultarían alterados como consecuencia de los eventos asociados a la apnea obstructiva del sueño y que estarían relacionados con la presencia de disfunción eréctil. Tampoco se ha evaluado el impacto del tratamiento con CPAP en dichos marcadores y en la prevalencia de disfunción eréctil en el paciente SAOS. Estos serán los objetivos principales del presente estudio.

MEMORIA DE SOLICITUD DEL PROYECTO DE INVESTIGACIÓN

Investigador principal: Mercè Pascual Queralt

Bibliografía más relevante (Máximo 1 página)

(1) Young T, Palta M, Dempsey J et al. The occurrence of sleep-disordered breathing among middle-aged adults. N Engl J Med 1993; 328:1230-1235.

(2) Phillipson EA. Sleep Apnea-a major public health problem. N Engl J Med 1993; 328(17):1271-1273.

(3) Mehra R, Principe-Rodriguez K, Kirchner HL et al. Sleep apnea in acute coronary syndrome: high prevalence but low impact on 6-month outcome. Sleep Med 2006; 7(6):521-528.

(4) Gami AS, Howard DE, Olson EJ et al. Day-night pattern of sudden death in obstructive sleep apnea. N Engl J Med 2005; 352(12):1206-1214.

(5) Kuniyoshi FH, Garcia-Touchard A, Gami AS et al. Day-night variation of acute myocardial infarction in obstructive sleep apnea. J Am Coll Cardiol 2008; 52(5):343-346.

(6) Bradley TD, Floras JS. Obstructive sleep apnoea and its cardiovascular consequences. Lancet 2009; 373(9657):82-93.

(7) Mooe T, Franklin KA, Wiklund U et al. Sleep-disordered breathing and myocardial ischemia in patients with coronary artery disease. Chest 2000; 117(6):1597-1602.

(8) Lue TF, Tanagho EA. Physiology of erection and pharmacological management of impotence. J Urol. 1987 May;137(5):829-36.

(9) Tal R, Mueller A, Mulhall JP. The correlation between intracavernosal pressure and cavernosal blood Oxygenation. J Sex Med. 2009 Oct;6(10):2722-7. Epub 2009 Aug 4.

(10) Brodereick G, Lue T. Evaluation a nonsurgical management of erectile dysfunction. Campbell's Urology 8º Ed. Cap 46.

(11) Feldman HA, Goldstein I, Hatzichristou DG, Krane RJ, McKinlay JB. Impotence and its medical and psychosocial correlates: results of the Massachusetts Male Aging Study. J Urol. 1994 Jan;151(1):54-61.

(12) Lyngdorf P and Hemmingsen L. Epidemiology of erectile dysfunction and its risk factors: a practice-based study in Denmark. International Journal of Impotence Research (2004) 16, 105 -111.

MEMORIA DE SOLICITUD DEL PROYECTO DE INVESTIGACIÓN

Investigador principal: Mercè Pascual Queralt

Hipótesis

Los mecanismos fisiopatológicos que resultan alterados como consecuencia de los eventos asociados a la apnea obstructiva del sueño (hipoxia-reoxigenación, arousals y fragmentación del sueño), están relacionados con un incremento en el riesgo de desarrollar disfunción eréctil en pacientes con Síndrome de Apnea Obstructiva del Sueño. La alteración en el perfil de expresión de marcadores biológicos (disfunción endotelial y trastorno cardiovascular, y hormonas sexuales) que resultan alterados como consecuencia de los eventos asociados al SAOS, se relacionan con el desarrollo de disfunción eréctil.

El tratamiento con presión de aire positiva continua (CPAP) revierte las repercusiones del SAOS y en pacientes con disfunción eréctil puede mejorar la función eréctil.

Objetivos

PRIMARIOS

- Determinar el perfil de síntesis de distintos marcadores biológicos relacionados con disfunción endotelial y trastorno cardiovascular, que resultan alterados como consecuencia del Síndrome de la Apnea Obstructiva del Sueño y su relación con el riesgo de desarrollar Disfunción Eréctil.
- Comparar el perfil de secreción de hormonas sexuales relacionadas con el control de la función eréctil en un grupo de pacientes con Síndrome de Apnea Obstructiva del Sueño con y sin Disfunción Eréctil.
- Evaluar el impacto del tratamiento con CPAP sobre los marcadores relacionados con disfunción eréctil.

SECUNDARIOS

- Evaluar la prevalencia de Disfunción Eréctil en pacientes con Síndrome de Apnea Obstructiva del Sueño
- Comparar el perfil psicológico de pacientes con Síndrome de Apnea Obstructiva del Sueño con y sin Disfunción Eréctil con el objetivo de detectar trastornos psicológicos relacionados con el riesgo de desarrollar Disfunción Eréctil.

Ajustarse al espacio disponible

MEMORIA DE SOLICITUD DEL PROYECTO DE INVESTIGACIÓN

Investigador principal: Mercè Pascual Queralt

Metodología (Diseño, sujetos de estudio, variables, recogida y análisis de datos y limitaciones del estudio)
(Máximo 3 páginas)

1. ASPECTOS GENERALES:
Estudio multicéntrico en el que participa el Hospital Arnau de Vilanova y el Hospital Santa María de Lleida. Todos los sujetos participantes en este estudio serán reclutados de forma consecutiva de entre aquellos que atiendan a la Unidad del Sueño mencionadas por razones asistenciales. El diagnóstico de SAOS se basará en el resultado de una polisomnografía completa, siguiendo la normativa de la Sociedad Española de Neumología y Cirugía Torácica (SEPAR) (Montserrat et al. Arch Bronconeumol 1998). Las definiciones y estadiaje de los estudios polisomnográficos se realizarán asimismo de acuerdo con dicha normativa. Todos los sujetos incluidos (como pacientes) deben cumplir las indicaciones de tratamiento con CPAP de acuerdo con las directrices de SEPAR.

- Primera fase: Estudio epidemiológico piloto transversal.
- Segunda fase: Estudio epidemiológico longitudinal cuasi-experimental prospectivo.

2. ASPECTOS ETICOS
Se informará al paciente por escrito de la naturaleza y propósitos del estudio. Los derechos de los pacientes estarán en todo momento protegidos por la declaración de Helsinki. Se solicitará consentimiento informado por escrito a todos los participantes en el estudio. Este estudio ha sido aprobado por el Comité de Ética e Investigación Clínica de nuestro Hospital.
El responsable de la base de datos será del investigador principal (Mercè Pascual). Dicha base de datos se ubicará en el ordenador de Mercè Pascual, que se encuentra situado en el despacho de Urología de la segunda planta del Hospital Santa María.
La base de datos se realizará en formato Excel. En ella se recogerán todas las variables clínicas, analíticas, polisomnográficas y de exploraciones en relación con el estudio. La identificación del paciente en la base de datos se realizará por medio de un código asignado. La relación entre el código del estudio y los datos de filiación y clínicos del paciente quedarán registrados en un documento al que solo tendrá acceso Mercè Pascual.
Las muestras biológicas pertenecientes a los pacientes se localizarán en un congelador, destinado a este fin, situado en la Unidad de Investigación perteneciente al IRBLleida localizado en el Hospital Universitario Arnau de Vilanova. La identificación de las muestras se realizará por medio un código que se corresponde con el código del estudio asignado al paciente.

3. TAMAÑO MUESTRAL

Al aceptar un riesgo alfa de 0.05 y un riesgo beta de 0.2 en una prueba bilateral, son necesarios 63 sujetos en cada brazo de estudio para reconocer como estadísticamente significativa una diferencia de 2 unidades en la función eréctil (puntuación IIEF15) después de la intervención. La diferencia mínima clínicamente importante de 2 unidades se define de acuerdo con los resultados de Rosen et al. para pacientes con disfunción eréctil leve. Se estima una desviación estándar común de 4 unidades. Suponiendo un 69% de prevalencia de disfunción eréctil entre los pacientes con SAOS de acuerdo a la literatura previa, 183 pacientes con SAOS deben ser evaluados para obtener los 126 pacientes con DE requeridos.

4. POBLACION Y METODOS.
Se estudiarán dos poblaciones de sujetos: pacientes SAOS con disfunción eréctil y pacientes SAOS sin disfunción eréctil. De forma específica la inclusión de pacientes se va a estratificar por las variables potencialmente confundentes: índice de masa corporal (IMC), y edad.

a) Grupo SAOS con disfunción eréctil: Se incluirán en total 80 pacientes consecutivos que cumplan los criterios que serán definidos posteriormente;

i) Criterios de Inclusión: Varones entre 18 y 70 años, diagnóstico de SAHS (índice de apnea-hipopnea > 10 h-1, evaluado por polisomnografía), diagnóstico de disfunción eréctil (IIEF<25), y firma del consentimiento informado.

ii) Criterios de exclusión

-Incapacidad psico-física para realizar los cuestionarios o colaborar con la realización de las pruebas.

-Pacientes que presenten alguna de las siguientes condiciones: Vasculogénicas: Enfermedad cardiovascular; Neurogénicas: Esclerosis múltiple, Enfermedad de Parkinson, discopatía vertebral; Antecedentes de cirugía pélvica o retroperitoneal; Malformaciones congénitas o adquiridas: Enfermedad de Peyroné, hipospadias, epispadias, fractura peneana; Alteraciones hormonales: hipogonadismo, hiperprolactinemia, hiper o hipotiroidismo, enfermedad de Cushing; Consumo de drogas: enolismo, cocaína, heroína; Tratamiento con alguno de los siguientes fármacos: antidepresivos, antipsicóticos.
b) Grupo SAOS sin disfunción eréctil: Se incluirán en total 150 pacientes consecutivos que cumplan los mismos criterios que el grupo anterior excepto el punto de los criterios de inclusión. Se define paciente sin disfunción eréctil aquel que presente un IIEF 26-30.
Se reclutarán de forma consecutiva todos los pacientes remitidos a la Unidad del Sueño del Hospital Arnau de Vilanova - Hospital de Santa María para descartar Síndrome de Apnea Obstructiva del Sueño mediante la realización de una polisomnografía y que cumplan los criterios de inclusión y no cumplan los de exclusión.

5. INSTRUMENTOS DE MEDIDA Y VARIABLES DE ESTUDIO:
- Hábitos tóxicos. Variables clínicas y antropométricas. Historia clínica detallada. Se registrarán los datos de edad, índice de masa corporal, tensión arterial, circunferencia de cadera, circunferencia de cintura y circunferencia de cuello.
- Determinaciones biológicas: Las muestras de sangre se extraerán a las 8 de la mañana (10 ml en tubo sin anticoagulante y 7 ml en tubo con EDTA), se centrifugarán (3000 rpm, 20 minutos, 4ºC) y el suero y plasma obtenidos serán alicuotados y almacenados a -80ºC hasta el momento del análisis. Las muestras serán analizadas en duplicado en la misma serie analítica. Las muestras serán enviadas al biobanco del IRB. Se determinará:
a) Perfil bioquímico básico que incluya glucosa, lípidos (triglicéridos, colesterol total, HDL colesterol, LDL colesterol).
b) Perfil Hormonal: testosterona, sHBG, prolactina, LH, FSH mediante RIA (R&D Systems, UK)
c) Cuantificación de óxido nítrico, factores de hipercoagulabilidad, marcadores de disfunción endotelial (ADMA, ICAM-1, VCAM-1, VEGF) mediante RIA (R&D Systems, UK)
- Variables funcionales:
i) Polisomnografía convencional: Se realizará según recomendaciones internacionales. El tiempo mínimo de registro será de 6 horas y el tiempo mínimo de sueño para que se considere válido será de 3 horas. El análisis se realizará de acuerdo con las recomendaciones del Consenso Nacional sobre el síndrome de apneas-hipopneas del sueño. Se considera apnea obstructiva la ausencia o reducción > 90% de la señal respiratoria de > 10 segundos de duración en presencia de esfuerzo respiratorio detectado por las bandas toracoabdominales. Se considera hipopnea una reducción discernible (>30% y < 90%) de la amplitud de la señal respiratoria de > 10 segundos de duración que se acompaña de una desaturación (>3%) y/o micro-despertar en el electroencefalograma. El número de apneas más hipopneas dividido por las horas de sueño es el índice de apnea-hipopnea (IAH). El síndrome obstructivo de apnea-hipopnea se define por un IAH > o igual a 10, con síntomas de excesiva somnolencia diurna. SAHS leves se define por un IAH de 10-14; moderado, 15-30; y severo > 30.
ii) Test Epworth: El grado de somnolencia auto-percibida se analizará mediante la escala de somnolencia Epworth (ESS). Definición de somnolencia diurna excesiva (EDS): Se define la presencia de EDS cuando se obtiene un valor de escala del test de Epworth superior o igual a 10 y ausencia de EDS con un valor < 10. Esta diferenciación es importante ya que desde un punto de vista ético el paciente con SAHS que presenta un test de Epworth superior o igual a 10 debe seguir tratamiento con CPAP.
iii) Pulsioximetría: El registro continuo de la saturación de oxígeno durante la noche. Se evaluará el tiempo con una saturación < 90%, la saturación mínima y la saturación media.
iv) Test IIEF (International Index Erectile Function): Test que ha sido validado en 10 idiomas, entre ellos el español. Permite una evaluación de variables de forma continua. Valora la función sexual ampliamente abarcando: la función eréctil, la función orgásmica, el deseo sexual, la satisfacción sexual y la satisfacción general. Según los resultados del test se divide como disfunción eréctil: 6-10 Severa, 11-16 Moderada, 17-25 Leve, 26-30 Sin Disfunción Eréctil.
v) Test psicológico: SEAR (versión española), específico para Disfunción Eréctil, es autoadministrado y se diseñó para evaluar de forma específica aspectos psicosociales asociados a la Disfunción Eréctil. Consta de 14 ítems, con 5 posibles opciones de respuesta, mediante los que se evalúan con respecto a las últimas 4 semanas las siguientes 2 dimensiones: Relaciones sexuales (ítems 1-8) y Autoconfianza (9- 14), que a su vez se divide en 2 subdimensiones: Autoestima (ítems 9-12) y Relaciones en General (ítems 13 y 14). Finalmente, el cuestionario evalúa el grado de satisfacción del paciente con su función eréctil en general con respecto a las últimas 4 semanas (ítem final). El resultado se obtiene mediante la suma de las puntuaciones obtenidas en

cada ítem y su posterior transformación a una escala de 0 a 100, en la que mayores puntuaciones indican mejor calidad de vida.

4. Procesamiento de los datos y análisis estadístico:

i) Procesamiento: El análisis se llevará a cabo con el paquete de análisis estadístico SPSS (Versión 17.0)

ii) Análisis estadístico: En primer término, se realizará una depuración de la base de datos. Todas las variables del estudio se tabularán en una base de datos sólo accesible a los investigadores del estudio. Para la identificación de los pacientes se realizará la asignación de un código específico único. La clave de conexión entre el código y el sujeto está en manos del investigador principal que será quien se encargue de protegerlo y preservar la confidencialidad. El mismo, incluirá este código y su relación con el paciente en una base de datos almacenada en un lugar debidamente protegido y que sólo estará accesible al investigador principal del proyecto.

Estudio univariado: En el caso de las variables cualitativas se determinará su frecuencia y porcentaje válido. Se tendrán en cuenta los valores perdidos para dar los resultados. Para las variables cuantitativas se incluirán medidas de tendencia central, medidas de posición y de dispersión. Se realizará un estudio de normalidad para todas las variables numéricas.

<u>1ª Fase: Relación SAHS - DE (Estudio exploratorio, transversal)</u>

Para la comparación entre los dos grupos (SAHS-Controles), variables cualitativas, se empleará X^2 o Test exacto de Fisher en el caso de frecuencias esperadas <5. En el caso de las variables cuantitativas, Test T-Student o U-MannWithney en el caso que no se asuma normalidad.

Se tendrán en cuenta variables de confusión que ya están previamente descritas en la bibliografía para este tipo de relación (Edad, tabaquismo, HTA, DM, IMC). La interacción se determinará por la prueba de razón de verosimilitud considerando un nivel de significación <0.05. Se determinarán las Odds de prevalencia de en cada grupo en un modelo final multivariado de regresión logística.

<u>2ª Fase: Impacto de la CPAP en la DE (Estudio observacional prospectivo)</u>

Se analizará el impacto de la CPAP (pacientes SAHS en tratamiento con CPAP) en la DE en cuanto a marcadores biológicos de disfunción endotelial, nivel de hormonas sexuales y perfil psicológico de los pacientes. Se realizará un análisis pre-post de datos apareados. Grados de concordancia entre medidas. Finalmente se realizará un modelo multivariado final

En todas las pruebas se considerará significativa una p<0.05.
