# Supplementary material for: Erectile dysfunction in obstructive sleep apnea patients: A randomized trial on the effects of Continuous Positive Airway Pressure (CPAP)
Source: PLoS One. 2018 Aug 8;13(8):e0201930. doi: 10.1371/journal.pone.0201930 (PMC6082539; doi:10.1371/journal.pone.0201930)
Supplement: S2 Protocol — (PDF) [file pone.0201930.s005.pdf]

MEMORY OF APPLICATION OF THE RESEARCH PROJECT

TITLE: Impact of erectile dysfunction in Obstructive Sleep Apnea Syndrome

Principal investigator: Mercè Pascual Queralt

Overview (objectives and methodology of the project): (maximum 250 words)

Introduction: Obstructive (OSA) sleep apnea syndrome is a common process that affects 2-4% of the general population, causes an increase in sympathetic activity, changes in systemic blood pressure, and is associated with cardiovascular disease. These changes can impact the erection mechanisms. Today's date, the studies that relate erectile dysfunction with Obstructive Sleep Apnea (OSA) syndrome are epidemiological type.

Objectives: To determine the impact of OSAS in erectile dysfunction from the psychological point of view organic (alteration of hormone levels, loco-regional disorders, cardiovascular disorders) as both (the OSA patient psychological assessment presenting erectile dysfunction). To do this we are going to determine the profile of synthesis of biological markers associated with Endothelial dysfunction and cardiovascular disorder that are altered as a result of OSA and their relationship with the presence of erectile dysfunction. In addition, the profile of secretion of sexual hormones in order to determine whether there is a pattern altered in OSAS patients with erectile dysfunction, for OSAS patients without erectile dysfunction will be evaluated. One of the objectives of the present study will also assess the impact of treatment with CPAP (3 months) in the expression profile of the analyzed markers associated with Endothelial dysfunction.

Methodology: Prospective multicenter study involving the Hospital Arnau de Vilanova and Santa Maria Hospital of Lleida. Patients who come to the sleep unit for OSAS diagnosis using conventional polysomnography will include. In addition, you will be: Test Epworth, pulse oximetry, Basic biochemical profile, analysis of biological markers (Endothelial dysfunction and cardiovascular disorder, and sex hormones), test questionnaire SEAR and IIEF (International Index Erectile Function). All those with a diagnosis of OSAS, after receiving treatment with CPAP for 3 months will again be studied, in order to assess the impact of treatment with CPAP in the markers associated with erectile dysfunction in patients with OSA.

MEMORY OF APPLICATION OF THE RESEARCH PROJECT
**Principal investigator**: Mercè Pascual Queralt
Background and current status of the subject

Sleep apnea syndrome - (SAHS) sleep hypopnea is characterized by the presence of respiratory pauses totals (apneas) or partial (hypopnea), due to the collapse of the upper airway during sleep. It is a common process that affects 2-4% of the general population and is clinically characterized by the presence of snoring, daytime sleepiness, and deterioration in the quality of life (1). The index apnea-hypopnea (AHI) represents the number of apneas or hypopneas per hour of sleep and is the usual way of defining the disease. Treatment consists of changes in lifestyle such as weight loss and applying pressure by nasal continuous positive (CPAP) during sleep. CPAP is an effective treatment that corrects the obstructive respiratory events, improve symptoms and quality of life of patients suffering from obstructive (OSA) sleep apnea syndrome. Each episode of apnea is accompanied by a decrease in arterial oxygen saturation, which quickly normalize with next ventilation (hypoxia - reoxygenation), sudden changes in the intrapleural pressure. It has shown that as it progresses the night is produced an increase in the number and length of apneas, increasing the degree of oxygen desaturation and intrathoracic pressure levels generated in each apnea (1,2,3). Each apneic event ends with an arousal (Electroencephalographic awakening). Episodes of intermittent hypoxia and the arousals cause an increase in sympathetic activity and sudden changes in systemic blood pressure, that contribute to the development of hypertrophy, myocardial, cardiac arrhythmias and death. Currently, the OSAS is considered as the main cause of secondary hypertension, is associated with cardiovascular disease and a relevant health problem.

In addition, repeated episodes of hypoxia - reoxygenation, and the increase in sympathetic activity can activate different pathogenic pathways that promote atherogenesis are: oxidative stress, Endothelial dysfunction, hypercoagulability and alterations metabolic such as insulin resistance. These physiological changes would explain the increased risk of developing cardiovascular disease in patients with OSAS (4-7).

Mechanisms of erection, a phenomenon neurovascular under hormonal control, including arterial dilation and relaxation of the smooth musculature and activation of venous-occlusive bodies system may be altered between the Affectations of the vascular system cavernous (8). In a physiological way occur spontaneous erections during sleep. Its purpose is the oxygenate the erectile tissue to maintain its vitality. Know that to a full erection is when the cavernous tissue has a greater oxygenation, but there are studies that suggest that a low intra-cavernous pressure, or partial erections, also allow a sufficient oxygenation of the erectile tissue and prevent the damage caused by hypoxia (9).

Erectile dysfunction is the inability to achieve and maintain one erection sufficient for satisfactory sexual intercourse for at least three months, except in the case of trauma or surgery (10). Although it is a benign disorder, affects also the patient psychosocial health and has one significant impact on the quality of life of patients as your partner and family environment (11). A worldwide several epidemiological studies have been conducted to determine the prevalence of erectile dysfunction, which has varied between 12% - 71% depending on the methodology, the study group, the sample size and the used definition of erectile dysfunction. Also performed studies of relationship between erectile dysfunction and diseases such as hypertension, cardiovascular disease, Dyslipidemia, diabetes and depression (12). There are several factors that

can explain the origin of erectile dysfunction. Factors of cardiovascular risk, neuroendocrine disorders, risk factors psychological and local organic alterations secondary to regional surgeries, birth defects or acquired malformations.

Today's date, studies that relate erectile dysfunction with OSAS are epidemiological, based on cross-sectional descriptive studies, which include many variables and where there are multiple confounding factors. At the same time, many patients with OSAS are chronic snorers, thing that can lead to a worse break both patient and partner and a lower yield in sexual activity. No previous study has made an assessment of the biological markers (cardiovascular markers and sex hormones) that would be altered as a consequence of the events associated with obstructive sleep apnea and that would be related with the presence of erectile dysfunction. Neither has been evaluated the impact of treatment with CPAP in these markers and the prevalence of erectile dysfunction in the OSA patient. These are the main objectives of the present study.

MEMORY OF APPLICATION OF THE RESEARCH PROJECT
**Principal investigator**: Mercè Pascual Queralt

Most relevant bibliography (maximum 1 page)

(1) Young T, Palta M, Dempsey J et to the. The occurrence of sleep-disordered breathing among middle-aged adults. N Engl J Med 1993; 328:1230-1235.

(2) Phillipson EA. Sleep Apnea-a major public health problem. N Engl J Med 1993; 328 (17): 1271-1273.

(3) r. Mehra, Principe-Rodriguez K, Kirchner HL et to the. Sleep apnea in acute coronary syndrome: high prevalence but low impact on 6-month outcome. Sleep Med 2006; 7 (6): 521-528.

(4) Gami AS, Howard, Olson EJ et to the. Day-night pattern of sudden death in obstructive sleep apnea. N Engl J Med 2005; 352 (12): 1206-1214.

(5) Kuniyoshi FH, García-Touchard, Gami AS et to the. Day-night variation of acute myocardial infarction in obstructive sleep apnea. J Am Coll Cardiol 2008; 52 (5): 343-346.

(6) Bradley TD, Floras JS. Obstructive sleep apnoea and its cardiovascular consequences. Lancet 2009; 373 (9657): 82-93.

(7) Mooe T, Franklin KA, Wiklund U et to the. Sleep-disordered breathing and myocardial ischemia in patients with coronary artery disease. Chest 2000; 117 (6): 1597-1602.

(8) Lue TF, Tanagho EA. Physiology of erection and pharmacological management of impotence. J Urol. 1987 May; 137 (5): 829-36.

(9) such R, Mueller A, Mulhall JP. The correlation between intracavernosal pressure and cavernosal blood Oxygenation. J Sex Med. 2009 Oct; 6 (10): 2722-7. EPUB 2009 Aug 4.

(10) Brodereick G, Lue T. Evaluation to nonsurgical management of erectile dysfunction. Campbell's Urology 8th Ed. 46 Cap.

(11) Feldman HA, Goldstein I, Hatzichristou DG, Krane RJ, McKinlay JB. Impotence and its medical and psychosocial correlates: results of the Massachusetts Male Aging Study. J Urol. 1994 jan; 151 (1): 54-61.

(12) Lyngdorf P and Hemmingsen L. Epidemiology of erectile dysfunction and its risk factors: a practice-based study in Denmark. International Journal of Impotence Research (2004) 16, 105-111.

MEMORY OF APPLICATION OF THE RESEARCH PROJECT

Principal investigator: Mercè Pascual Queralt

Hypothesis

The pathophysiological mechanisms that are altered as a result of the events associated with obstructive apnea of the dream (hypoxia - reoxygenation, arousals and sleep fragmentation), are related to an increase in the risk of developing erectile dysfunction in patients with Obstructive Sleep Apnea Syndrome. Alteration in expression profiling of biological markers (Endothelial dysfunction and cardiovascular disorder, and sex hormones) that are altered as a result of the events associated with OSA, are related to the development of erectile dysfunction.

Positive air pressure (CPAP) continuous therapy reverses the impact of OSAS and in patients with erectile dysfunction can improve erectile function.

Objectives

PRIMARY

- To determine the profile of synthesis of various biological markers associated with Endothelial dysfunction and cardiovascular disorder, which are altered as a result of the obstructive Sleep Apnea Syndrome and its relationship to the risk of developing Erectile dysfunction.
- Compare the profile of secretion of sexual hormones related to control of erectile function in a group of patients with Obstructive Sleep Apnea Syndrome with and without erectile dysfunction.
- Assess the impact of treatment with CPAP on markers related to erectile dysfunction.

SECONDARY

- Evaluate the prevalence of erectile dysfunction in patients with Obstructive Sleep Apnea Syndrome
- Compare the psychological profile of patients with Obstructive Sleep Apnea Syndrome with and without erectile dysfunction in order to detect psychological disorders related to the risk of developing erectile dysfunction.

MEMORY OF APPLICATION OF THE RESEARCH PROJECT

Principal investigator: Mercè Pascual Queralt

Methodology (subjects of study, variables, collection and analysis of data and limitations of the study design) (Maximum 3 pages)

1. GENERAL ASPECTS:

Multicenter study involving the Hospital Arnau de Vilanova and the Santa Maria Hospital of Lleida. All the participants in this study will be recruited consecutively from among those who attend the sleep Unit mentioned for welfare reasons. The diagnosis of OSA will be based on the result of a polysomnogram full, according to the regulations of the Spanish society of Pneumology and thoracic surgery (SEPAR) (Montserrat et al. Arch Bronconeumol 1998). Definitions and staging of the Polysomnographic will also be in accordance with such regulations. All subjects including (as patients) must meet indications for treatment with CPAP according to the guidelines of SEPAR.

- <u>First phase</u>: cross-sectional pilot epidemiological study.

- <u>Second phase</u>: quasi-experimental prospective longitudinal epidemiological study.

2. ETHICAL ASPECTS

The patient will be informed in writing of the nature and study purposes. The rights of patients will be at all times protected by the Declaration of Helsinki. Informed written consent will be requested to all participants in the study. This study has been approved by the Committee of ethics and clinical research of our Hospital.
Responsible for the database will be the principal investigator (Merce Pascual). That database will be located on the computer of Merce Pascual, Maria located in urology at the second floor of the Hospital Holy Office.
The data base will be held in Excel format. It will include all the clinical, analytical, Polysomnographic and scans in relation to the study. The identification of the patient in the database will be made by means of a code. The relationship between the code of the study and affiliation data and clinical of the patient will be recorded in a document that will only have access Merce Pascual.
The biological samples from patients will be located in a freezer, earmarked for this purpose, located in the research unit belonging to the IRBLleida located in the Arnau de Vilanova University Hospital. Identification of the samples will take place by half a code which corresponds to the code of the study assigned to the patient.

3. SAMPLE SIZE

By accepting a 0.05 Alpha risk and risk in a bilateral test 0.2 beta, 63 subjects in each study arm are required to recognize as statistically significant a difference of 2 units in the erectile function (IIEF15 score) after the intervention. The minimal clinically important difference of 2 units is defined in accordance with the results of Rosen et to the. for patients with mild erectile dysfunction. It is estimated a common standard deviation of 4 units. Assuming a 69% prevalence of erectile dysfunction among patients with OSAS according to previous literature, 183 patients with OSAS must be evaluated for 126 patients with of required.

4 POPULATION AND METHODS

Two populations of subjects will be studied: OSAS patients with erectile dysfunction and OSAS patients without erectile dysfunction. Specifically, the inclusion of patients is to stratify the variables potentially confounders: (BMI) body mass index, and age.

a) OSA group with erectile dysfunction: 80 consecutive patients who meet the criteria which will be defined later; will be included in total
(i) Inclusion criteria: men between 18 and 70 years old, diagnosis of SAHS (> 10 h-1, evaluated by polysomnography apnea-hypopnea index), diagnosis of erectile dysfunction (IIEF < 25), and signature of informed consent.
II) exclusion criteria
-Psycho-physical disability to make questionnaires or collaborate with the testing.
-Patients who have any of the following conditions: cardiovascular disease; Neurogenic: Multiple sclerosis, Parkinson's disease, vertebral discopathy; History of pelvic surgery or retroperitoneal; Acquired or congenital malformations: Peyrone disease, hypospadias, epispadias disease, fracture penile; Hormonal abnormalities: Hypogonadism, hyperprolactinemia, hyper or hypothyroidism, Cushing's disease; Drug abuse: alcoholism, cocaine, heroin; Treatment with any of the following drugs: antidepressants, antipsychotics.

b) Group OSA without erectile dysfunction: 150 consecutive patients who meet the same criteria as the previous group except the point of inclusion criteria will be included in total. Without erectile dysfunction patient is defined who present an IIEF 26-30.

All patients referred to the unit of the dream of the Hospital Arnau de Vilanova - Santa Maria Hospital to rule out Obstructive Sleep Apnea Syndrome by means of a polysomnogram will be recruited consecutively and that they fulfil the criteria of inclusion and not meet those of exclusion.

5. MEASUREMENT INSTRUMENTS AND STUDY VARIABLES

-Toxic habits. Clinical and anthropometric variables. Detailed medical history. Recorded data for age, body mass, blood pressure, waist circumference and hip circumference, neck circumference.

-Biological determinations: blood samples will be drawn at 8 in the morning (10 ml in tube without anticoagulant) and 7 ml in EDTA tube, is centrifuged (3000 rpm, 20 minutes, 4 ° C) and serum and plasma obtained will be aliquoted and stored at - 80 ° C so far d the analysis. The samples will be analyzed in duplicate on the same analytical series. Samples will be sent to the IRB BioBank. Shall be determined:
a) Basic biochemical profile that includes glucose, lipids (triglycerides, total cholesterol, HDL cholesterol, LDL cholesterol).
b) Hormonal profile: testosterone, sHBG, prolactin, LH and FSH by RIA (R & D Systems, UK)
(c) quantification of nitric oxide, hypercoagulability factors, markers of Endothelial dysfunction (ADMA, ICAM-1, VCAM-1, VEGF) by RIA (R & D Systems, UK)

-Functional variables:
(i) conventional polysomnography: will be held according to international recommendations. The minimum registration time will be 6 hours and the minimum time of dream to be considered valid shall be 3 hours. The analysis will be done in accordance with the recommendations of the national consensus on sleep apnea-hypopnea syndrome. Obstructive sleep apnea the absence or reduction is considered >

90% of the respiratory signal for > 10 seconds of duration in the presence of respiratory effort detected by the Thoracoabdominal bands. Hypopnea is a discernible reduction (> 30% and < 90%) of the amplitude of the respiratory signal for > 10 seconds of duration that is accompanied by a desaturation (> 3%) or micro-awakenings on the electroencephalogram. The number of apneas more hypopneas divided by the hours of sleep is the index of apnea (IAH). Obstructive sleep apnea-hypopnea syndrome is defined by an AHI > or equal to 10, with symptoms of excessive daytime sleepiness. Mild OSAS is defined by an AHI of 10-14; moderate, 15-30; and severe > 30.
II) Test Epworth: the degree of sleepiness auto-perceived will be analyzed using the scale of sleepiness Epworth (ESS). Definition of excessive daytime sleepiness (EDS): the presence of EDS is defined when you get a scale value greater than or equal to 10 Epworth test and absence of EDS with a value < 10. This differentiation is important since from an ethical point of view SAHS patient presenting a greater than or equal to 10 Epworth test must follow treatment with CPAP.
(iii) pulse oximetry: continuous recording of the saturation of oxygen during the night. It will evaluate the time with a saturation < 90%, the minimum saturation and media saturation.
(iv) Test IIEF (International Index Erectile Function): Test that the Spanish has been validated in 10 languages, among them. It allows an evaluation of variables continuously. Valued sexual function broadly encompassing: erectile function, Orgasmic function, sexual desire, sexual satisfaction and overall satisfaction. According to the results of the test is divided as erectile dysfunction: 6-10 severe, 11-16 moderate, minor 17-25, 26-30 without erectile dysfunction.
(v) psychological Test: SEAR (Spanish version), specific for erectile dysfunction, it is self-administered and was designed to evaluate specific aspects associated with erectile dysfunction psychosocial. Consists of 14 items, with 5 possible response options, which are evaluated with respect to the last 4 weeks the following 2 dimensions: (items 1-8) sex and self-confidence (9-14), which in turn is divided into 2 subdimensiones: self-esteem (items (9-12) and relationships in General (items 13 and 14). Finally, the questionnaire evaluates the degree of patient satisfaction with their erectile function in general over the past 4 weeks (final item). The result is obtained by the sum of the scores obtained in each item and its subsequent transformation on a scale from 0 to 100, where higher scores indicate better quality of life.

Processing of data and statistical analysis:
(i) processing: the analysis will be carried out with the statistical analysis SPSS (Version 17.0) package
(ii) statistical analysis: first, a purge of the database will be held. All the variables of the study were tabulated in a database accessible only to the researchers of the study. For the identification of the patients will be the allocation of a specific code. The key connection between the code and the subject is in the hands of the principal investigator who will be who is responsible for protecting it and preserve confidentiality. It will include this code and its relationship with the patient in a database stored in a properly protected place and that will only be accessible to the principal investigator of the project.
Univariate study: qualitative variables should be determined their frequency and percentage valid. The lost values shall be taken into account to give the results. For quantitative variables include measures of central tendency, measures of position and dispersion. You will carry out a study of normalcy for all numeric variables.

1st phase: relationship SAHS - (exploratory, cross-sectional study)
For the comparison between the two groups (SAHS - Controls), qualitative variables, *X* will be used*2* or in the case of expected frequencies Fisher's exact Test < In the case of quantitative variables, T-Student Test, or U-MannWithney in the case that are not assumed normality.

Confusion variables that are previously described in the literature for this type of relationship (age, smoking, ETS, DM, IMC) shall be taken into account. The interaction is determined by the test of likelihood ratio whereas a significance level < 0.05. They will be determined the prevalence Odds of each group in a final multivariate logistic regression model.

Phase 2: impact of CPAP in the of (prospective observational study)
We will analyze the impact of the CPAP (OSAS patients treated with CPAP) in the of in terms of biological markers of Endothelial dysfunction, level of sex hormones and psychological profile of the patients. An analysis will be pre-post of paired data. Degree of concordance between measures. Finally held a final multivariate model.
All tests will be deemed significant a $p < 0.05$.
